# Dimensional Reduction for Single-Molecule Imaging of DNA and Nucleosome Condensation by Polyamines, HP1α and Ki-67

**DOI:** 10.1021/acs.jpcb.2c07011

**Published:** 2023-02-28

**Authors:** Nils A. Benning, Jacob Kæstel-Hansen, Fahad Rashid, Sangwoo Park, Raquel Merino Urteaga, Ting-Wei Liao, Jingzhou Hao, James M. Berger, Nikos S. Hatzakis, Taekjip Ha

**Affiliations:** † Department of Biology, Johns Hopkins University, Baltimore, Maryland 21218, United States; ‡ Department of Chemistry and Nanoscience Centre, University of Copenhagen, Copenhagen 2100, Denmark; § Department of Biophysics and Biophysical Chemistry, Johns Hopkins University School of Medicine, Baltimore, Maryland 21205, United States; ∥ Department of Biophysics, Johns Hopkins University, Baltimore, Maryland 21218, United States; ⊥ Novo Nordisk Foundation Centre for Protein Research, Faculty of Health and Medical Sciences, University of Copenhagen, Copenhagen 2100, Denmark; # Department of Biomedical Engineering, Johns Hopkins University, Baltimore, Maryland 21218, United States; ∇ Howard Hughes Medical Institute, Baltimore, Maryland 21205, United States

## Abstract

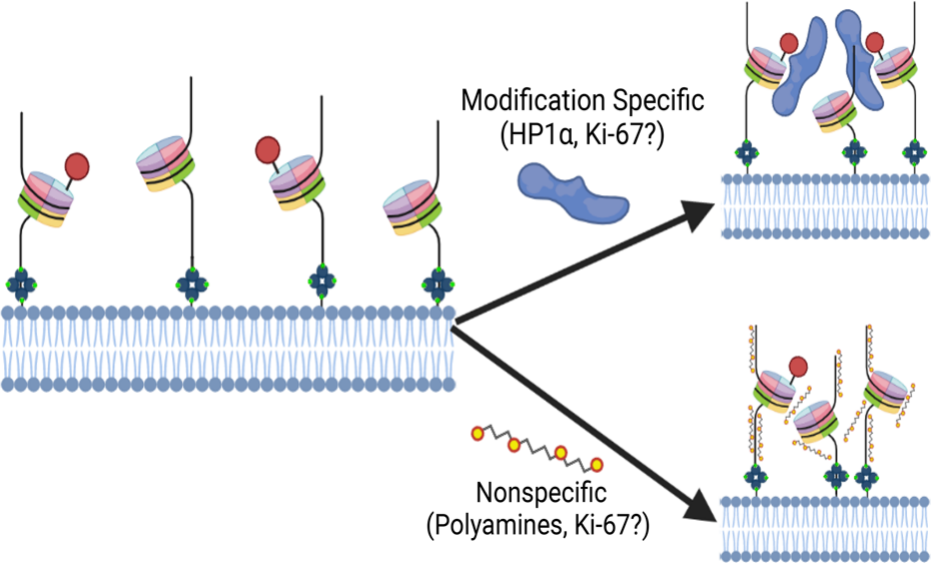

Macromolecules organize themselves into discrete membrane-less compartments. Mounting evidence has suggested that nucleosomes as well as DNA itself can undergo clustering or condensation to regulate genomic activity. Current in vitro condensation studies provide insight into the physical properties of condensates, such as surface tension and diffusion. However, methods that provide the resolution needed for complex kinetic studies of multicomponent condensation are desired. Here, we use a supported lipid bilayer platform in tandem with total internal reflection microscopy to observe the two-dimensional movement of DNA and nucleosomes at the single-molecule resolution. This dimensional reduction from three-dimensional studies allows us to observe the initial condensation events and dissolution of these early condensates in the presence of physiological condensing agents. Using polyamines, we observed that the initial condensation happens on a time scale of minutes while dissolution occurs within seconds upon charge inversion. Polyamine valency, DNA length, and GC content affect the threshold polyamine concentration for condensation. Protein-based nucleosome condensing agents, HP1α and Ki-67, have much lower threshold concentrations for condensation than charge-based condensing agents, with Ki-67 being the most effective, requiring as low as 100 pM for nucleosome condensation. In addition, we did not observe condensate dissolution even at the highest concentrations of HP1α and Ki-67 tested. We also introduce a two-color imaging scheme where nucleosomes of high density labeled in one color are used to demarcate condensate boundaries and identical nucleosomes of another color at low density can be tracked relative to the boundaries after Ki-67-mediated condensation. Our platform should enable the ultimate resolution of single molecules in condensation dynamics studies of chromatin components under defined physicochemical conditions.

## 1 Introduction

A resurgent concept in biology is the way cells organize biomolecules into membrane-less compartments through condensation. This organization facilitates cellular reactions and occurs once their concentration reaches a certain threshold. In the nucleus, these condensates facilitate processes such as DNA damage repair, gene repression, and ribosome biogenesis;^50−3^ in the cytosol, they facilitate mRNA processing, mRNA localization, translation, protein folding, along with many other processes.^54^ Dysregulation of condensates may result in the formation of pathological aggregates that lead to impaired cell function and may ultimately trigger cell death.^4−15^

Biomolecular condensation is promoted through interactions between macromolecules and depends on the valency of interactions. Entropy typically favors a heterogeneous mixture and is governed by environmental factors like pH and temperature, but many biological condensates circumvent this through multivalent interactions. Many proteins achieve multivalency through the interaction of two generally conserved modules: folded domains and low-complexity disordered segments.^2,5,16^ While recent advances detail the types of multivalent interactions and potential critical concentrations required for condensation, many studies lack the resolution required to distinguish phase-separated condensates from the formation of complex ordered structures.^17,18^ In addition, while live-cell-based studies have examined protein constructs with tunable valency,^19,20^ the kinetics of early condensate formation remain mostly unexplored.

Single-molecule studies provide enhanced resolution to observe the movement of individual macromolecules and how these mobile particles interact.^21,22^ Here, we used a supported lipid bilayer (SLB) to investigate two-dimensional movement of DNA and nucleosomes early in their condensation at the single-molecule level. Previously, SLBs have been used to study vesicle fusion, cell adhesion,^23−29^ and, importantly, the clustering of components within the bilayer like individual lipids and cholesterol.^30^ This platform allows us to correlate the kinetic behavior with condensate properties of SLB-bound molecules. Thus, it becomes possible to investigate the recruitment kinetics of other phase-separating proteins or macromolecules as we show for polyamines and HP1α. One protein of particular interest is Ki-67, a 2896 amino acid protein that helps maintain chromosome individuality by coating nucleosomes during mitosis.^37,38^ At the end of mitosis, Ki-67 aids in the exclusion of cytoplasm during reformation of the nuclear envelope. Thus, we used our experimental platform to examine Ki-67 as a condensing agent for DNA and nucleosomes.

## 2 Methods

### 2.1 Supported Lipid Bilayer (SLB) Generation

SLBs were generated based on studies that observe vesicle fusion into bilayers.^25^ Small unilamellar vesicles are prepared by drying a mixture of 93% POPC and 7% 18:1 biotinyl-PE (Avanti Polar Lipids, catalog #850457C and 870282C) under compressed nitrogen gas followed by overnight drying under vacuum. This “lipid cake” was hydrated with T50 Buffer (10 mM Tris-HCL pH 8.0, 50 mM NaCl) and pipetted several times to promote vesicle formation. After undergoing 15 freeze–thaw cycles using liquid nitrogen, small unilamellar vesicles (SUVs) were prepared by 21 passes through an extruder (Avanti) fitted with a 100 nm filter (Cytiva, catalog # 800309). SUVs were stored at 4 °C for up to 14 days.

### 2.2 Slide Preparation/Assembly

Quartz slides were cleaned by sonicating in methanol for 30 min, washing with acetone, and drying with nitrogen gas. After drying, the slides were sonicated in a 5% Alconox detergent solution for 30 min and rinsed with ddH_2_O. Slides were then sonicated in a 1 M KOH solution, rinsed with ddH_2_O, and burned using a propane torch. Glass coverslips were incubated in a 1× detergent solution (MP Biomedicals, 097667093) just below the boiling point for 1 h and then thoroughly rinsed with Milli-Q water. Glass coverslips were then baked in a furnace (Barnstead International, model FB1315M) at 540 °C, just below the melting point of the coverslip, for 5 h. Once clean, 1–2 mm strips of Scotch double-sided tape were placed between predrilled holes on the edges of the quartz slide. The glass coverslips were then briefly burned using a propane torch and placed on the tape-covered quartz slide. Excess tape was removed, and the edges of the slide were sealed using epoxy (Devcon), resulting in a final assembled reaction chamber. Functional imaging slides are made by injecting 20 μL of SUVs into a reaction chamber and incubating for 30 min to promote vesicle fusion and SLB formation. The excess free SUVs were then washed out with 200 μL of T50 Buffer.

### 2.3 TIRF Microscopy/Analysis

Fluorescently labeled macromolecules (DNA and mononucleosomes) were observed on the SLBs using objective-based total internal reflection fluorescence microscopy. The fluorescence emission was collected by oil objective (Nikon PlanApo, NA 1.40, 60×) and recorded by a back-illuminated electron-multiplying charge-coupled device camera (iXon3, Andor Technology) with a 50 ms exposure time.

To measure the initial condensation events, two criteria were put in place to define a potential condensate: no diffusion and an intensity value at least double that of an individual particle. Once a potential condensate is identified, other potential condensates can be easily located with the same criteria. When potential condensates are visually identified after moving the field of view to a random position or multiple condensates appear within one field of view, initial condensation has occurred.

Single-molecule tracking and fluorescence intensity analysis was performed using the ImageJ plugin, TrackMate, with a Laplacian of Gaussian (LoG) filter detector, which enables analysis of merging and splitting events. *XY* coordinates were obtained from these videos for each particle in each frame, and trajectories for each particle were analyzed using a Linear Assignment Problem (LAP) tracker.^31^ Diffusion coefficients for each particle were determined using a mean-squared displacement (MSD) analysis. Particles were assumed to undergo simple diffusion (where MSD = 4*Dt*), i.e., no confinement or directed movement. Trajectories were excluded when the trajectory length was fewer than 10 frames, indicating particles transiently entered the field of view. To track the real-time diffusional behavior, we utilized rolling MSD analysis by fitting MSD = 2*dDt*^α^, where *D* is the diffusional coefficient, α is a measure of persistence in the walk, and *d* is the dimension. For the analysis, we applied a system-specific threshold for restricted motion of MSD = 0.05 μm^2^.

### 2.4 Widefield Microscopy/Analysis

Bulk diffusion measurements were obtained by preparing SLBs as described above and imaging via a 555 nm excitation laser with a Cy3 emission filter using a water/oil-immersion objective (Nikon PlanApo λ, NA 0.75, 20×). FRAP experiments were performed using this microscope with a 50 mW bleaching laser at 405 nm and a Bruker Galvano mirror scanner. Regions of interest measuring 25 pixels in diameter on the SLB were bleached using 50% laser intensity for 1 s. Fluorescence recovery data was obtained immediately after bleaching and every 5 s up to 5 min.

### 2.5 Protein Purification and Nucleosome Assembly

HP1α was purified as described previously.^3^ Mononucleosomes with human histones were generated as described previously.^33^ Histones used in this study are free of modifications.

Full-length Ki-67 gene was synthesized (Twist Bioscience) and inserted into the yeast expression plasmid (-Ura) along with N-terminal hexahistidine tag by Gibson assembly. Ki-67 was expressed in the *S. cerevisiae* strain BCY123. Starter cultures were grown to saturation overnight in CSM-Ura- media supplemented with 2% dextrose, 2% lactic acid, and 1.5% glycerol at 30 °C. Starter cultures were then diluted 10-fold in YP media with 2% lactic acid and 1.5% glycerol and grown to an OD of 1.0–1.3 at 30 °C (12–15 h), at which point protein expression was induced by addition of 2% galactose for 6 h at 30 °C. Cells were harvested by centrifugation, resuspended in 1 mL of 1 mM EDTA and 250 mM NaCl per liter of culture, and flash frozen dropwise in liquid nitrogen for storage at −80 °C.

For purification, frozen pellets were lysed by cryogenic grinding in a freezer mill (SPEX SamplePrep). The cell powder was resuspended in K-Buffer (50 mM Hepes–KOH (pH 7.5), 300 mM NaCl, 30 mM imidazole (pH 8.0), 10% glycerol, 1 mM PMSF, 2.34 μM leupeptin, 1.45 μM pepstatin, and 0.5 mM TCEP). Lysate was clarified by centrifugation at 16 000 rpm in a JA 25.50 rotor for 45 min and loaded onto a HisTrap HP (GE) nickel-chelating Sepharose column. Protein was eluted in K-Buffer with 250 mM NaCl and 500 mM imidazole. Ki-67 fractions were loaded onto a Hitrap-SP column and eluted with K-buffer containing 1 M NaCl. Fractions containing Ki-67 were concentrated and loaded onto a Superose6 10/300gl column pre-equilibrated in storage buffer (50 mM Hepes-KOH (pH 7.5), 300 mM NaCl, 10% glycerol, 1 mM TCEP). Fractions containing Ki-67 were pooled, concentrated, and stored at −80 °C.

### 2.6 DNA Synthesis

DNA was synthesized from a Widom 601 sequence containing plasmid pGEMz_601 (Addgene, 26656) with primers containing a biotin and Cy3. AT-rich, GC-rich, and all primers were constructed by IDT.

125bp AT-rich DNA: 5′AGCGGTGATGCTGATAGAAGTATAATATTAATAATAAATTAAATATATTATATTAATAATTAATAATTAATAAATTAAAATATTATTTATAATAATTAAACATAATAGCTTCTGTGCGCC-3′

122bp GC-rich DNA: 5′TGAACCTGTACCCTTGTTGGCGCGTACGCGCGAACGCGTTATCGTCGCGTACGCGCGACGCGACGCGCGATCGCGAACGCGCGTCGTCGCGCGACGCGCGGCCTTGTAGATGAACTTGCG-3′

70bp DNA (−77N0): 5′CGTACGTGCGTTTAAGCGGTGCTAGAGCTGTCTACGACCAATTGAGCGGCCTCGGCACCGGGATTCTCCA-3′

147bp DNA (0N0): 5′CAGGATGTATATATCTGACACGTGCCTGGAGACTAGGGAGTAATCCCCTTGGCGGTTAAAACGCGGGGGACAGCGCGTACGTGCGTTTAAGCGGTGCTAGAGCTGTCTACGACCAATTGAGCGGCCTCGGCACCGGGATTCTCCA-3′

231bp DNA (43N43): 5′ACTATCCGACTGGCACCGGCAAGGTCGCTGTTCAATACATGCACAGGATGTATATATCTGACACGTGCCTGGAGACTAGGGAGTAATCCCCTTGGCGGTTAAAACGCGGGGGACAGCGCGTACGTGCGTTTAAGCGGTGCTAGAGCTGTCTACGACCAATTGAGCGGCCTCGGCACCGGGATTCTCCAGGGCGGCCGCGTATAGGGTCCATCACATAAGGGATGAACTCGG-3′

## 3 Results and Discussion

### 3.1 SLBs Provide a Platform for Measuring Fluid Properties and Condensates at a Single-Molecule Level

Supported lipid bilayers (SLBs) are a widely used platform that mimics the cellular membrane. SLBs can be integrated into many surface-based techniques and allow for investigating fundamental membrane biology.^25,26,30^ Recent work has shown that SLBs are a suitable platform for investigating membrane-bound liquid–liquid phase separation (LLPS), highlighting how traditional LLPS studies such as circular droplet formation and fusion can be replicated in two dimensions.^34^

As a proof of concept, we first formed SLBs containing 94% POPC, 1% 18:1 rhodamine PE for SLB visualization, and 5% phosphatidylethanolamine mimic containing a biotinylated headgroup, 18:1 Biotinyl PE, and bound a biotin-labeled double-stranded DNA to the SLB through an avidin linkage. After 43N43 DNA (N denotes the 147bp 601 Widom positioning sequence with 43 bp linker DNA) was bound on the SLB, widefield imaging was performed to confirm the fluorescence homogeneity across the surface of the SLB (Figure S1A). This was directly compared to SLBs containing 1% 18:1 rhodamine PE (99% POPC). A small area was selectively photobleached, and fluorescence recovery was observed with a halftime of 65 s for the DNA-bound SLB and 69 s for the SLB with no bound DNA (Figure S1B). From FRAP curves generated by the fluorescence recovery we determined the bulk diffusion coefficient of 0.59 ± 0.046 μm^2^/s for 18:1 rhodamine PE containing SLBs and 0.55 ± 0.044 μm^2^/s for 18:1 rhodamine PE-containing, DNA-bound SLBs. This indicates that the binding of DNA has little interference on the diffusion of lipids in SLBs. These values are also consistent with the diffusion coefficients of SLBs with membrane-bound molecules determined in other studies.^21^

**Figure 1:**
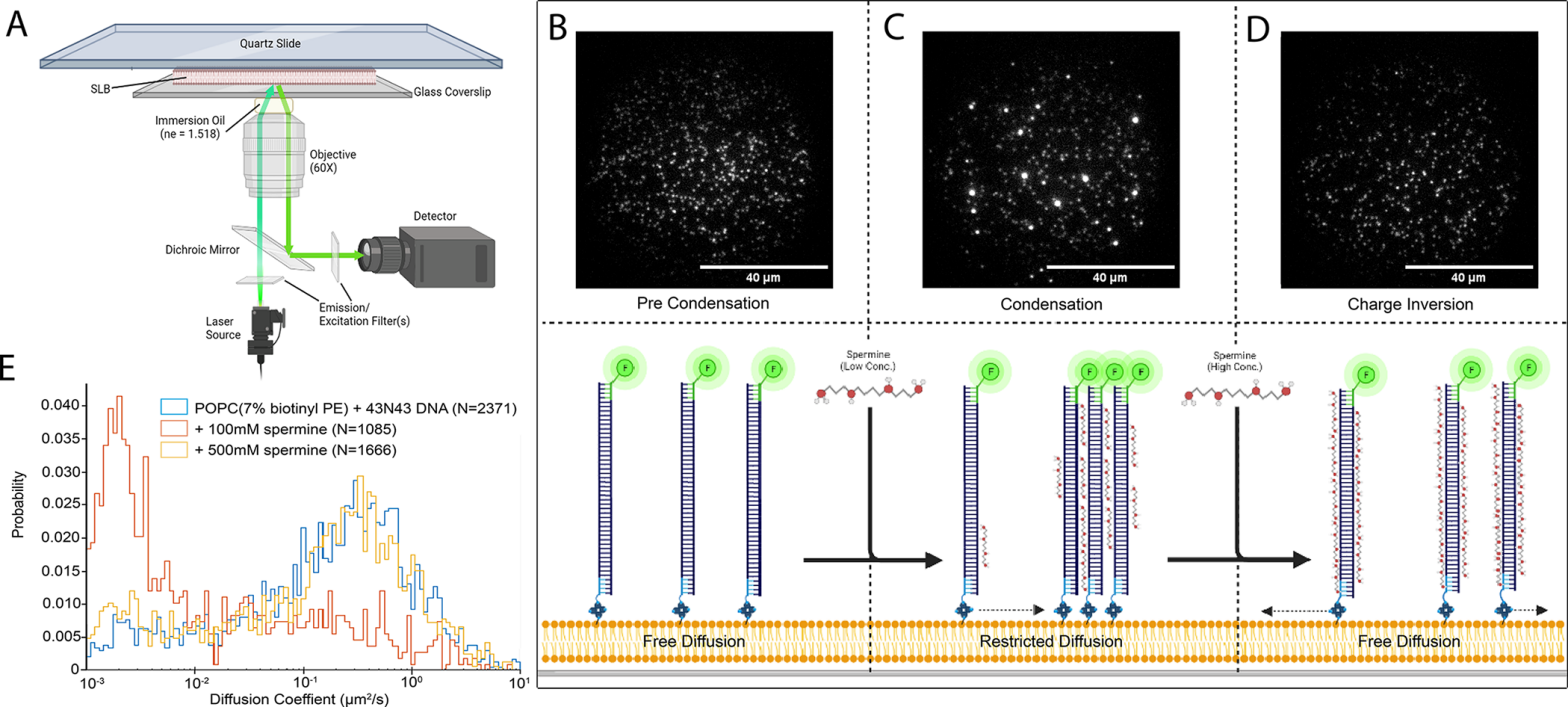
Experimental setup for experiments using objective-based total internal reflection fluorescence (oTIRF) microscopy in tandem with SLBs to observe spermine-mediated condensation and dissolution: (A) oTIRF microscope setup, (B) visual representation and oTIRF recording of DNA bound to 7% biotin containing SLB before addition of any condensing agent, (C) visual representation and oTIRF recording of DNA bound to 7% biotin containing SLB after promoting condensate formation through addition of 100 mM spermine, (D) visual representation and oTIRF recording of DNA bound to 7% biotin containing SLB after inducing charge inversion and condensate dispersal through addition of 500 mM spermine, and (E) diffusion histogram showing before, during, and after spermine-mediated condensation and charge inversion of 40N40 DNA.

By reducing the concentration of fluorescent molecules bound to the SLB, we could observe diffusion of single anchored DNA across the SLB surface (Figure 1A). The particle density is tuned to allow for individual particle tracking while maintaining a high enough density to allow for formation of small condensates (Figure 1B and 1C). To test the efficacy of SLBs as a platform for observing condensation, we first bound a 230 base pair (bp) long DNA labeled with Cy3 to SLBs. Lateral movement of particles was captured with a diverse population of diffusion coefficients (Figure 1B and 1E). We then added 100 mM spermine, a 4+ charged polyamine. Previously, spermine has been shown to bridge adjacent DNA duplexes^35^ to promote condensate formation. After the addition of spermine, individual particles formed into brighter small condensates (or clusters) (Figure 1C and 1E). In addition to the immobile condensates, we also observed mobile particles as well as arrested individual particles. The population of mobile particles is likely dependent on the concentration as well as the type of condensing agent: charge-based condensation as in the case of spermine leads to charge inversion-mediated condensate dispersal at sufficiently high concentrations. Condensate formation on SLB-bound particles relies on the lateral movement of phospholipids within the bilayer. Thus, irreversible membrane deformation or destruction may interfere with lateral movement, giving a false impression of condensation. To rule out this possibility, we induced charge inversion by increasing the concentration of spermine to 500 mM, where the negatively charged DNA backbones become coated in positive charges from spermine. The resulting charge inversion is observed as dispersal of condensates formed by spermine (Figure 1D and Video 1). Moreover, the mobility of the surface-bound particles recovers to levels before the addition of spermine (Figure 1D and 1E).

**Figure 2:**
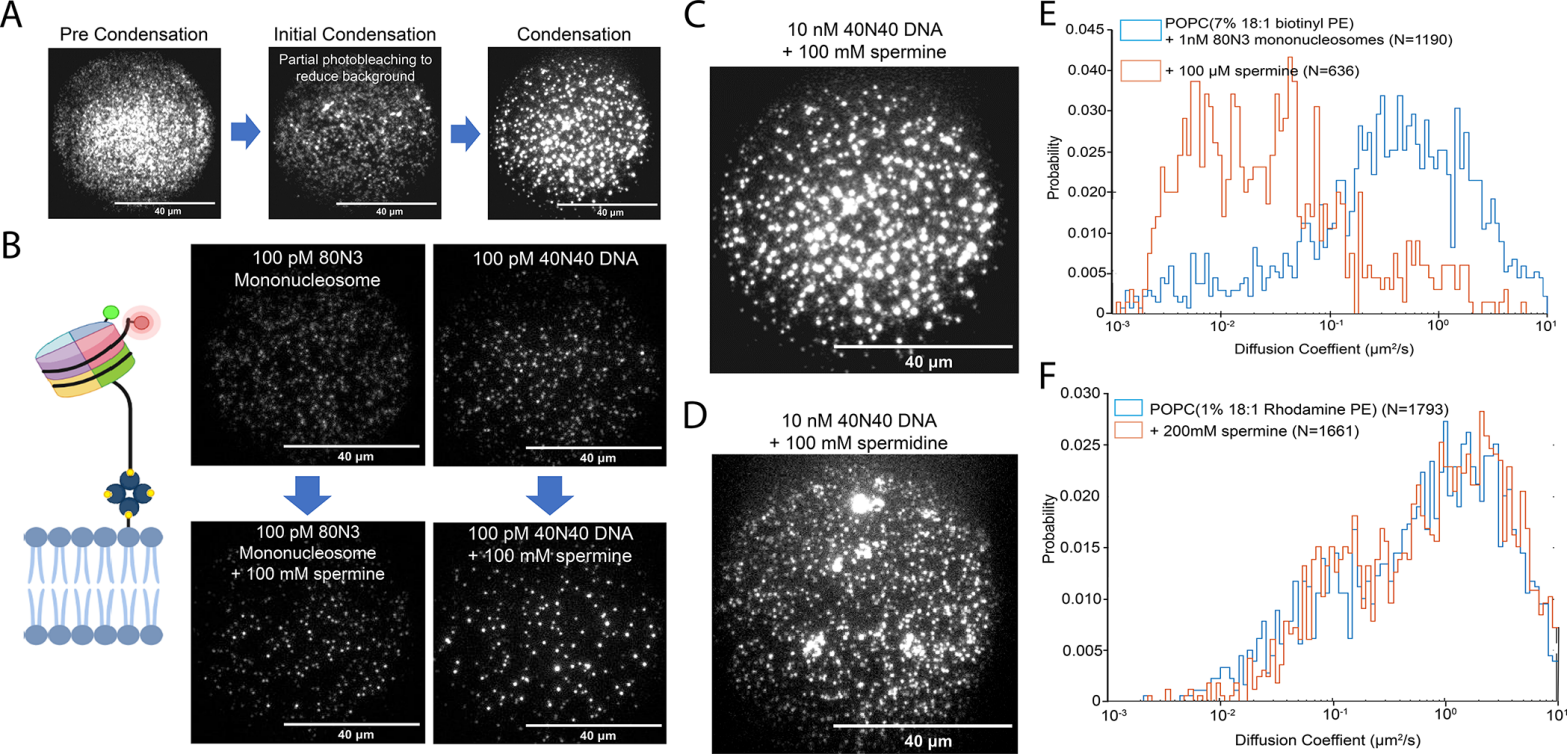
Condensation landscape. (A) Definitions associated with condensation as they are used in downstream experiments. (B) 80N3 mononucleosome construct used in subsequent experiments, and example recordings of 80N3 nucleosomes and 40N40 DNA before and after condensate formation by spermine. (C) SLB populated densely with DNA in the presence of spermine. (D) SLB populated densely with DNA in the presence of spermidine. (E) Diffusion histogram showing before and after spermine-mediated condensation of 80N3 nucleosomes. (F) Diffusion histogram of a noncondensing particle (rhodamine-conjugated lipid) in an SLB.

In this study, “initial condensation” is described as the formation of bright, immobile puncta early when many particles are still mobile and “definitive condensation” is defined by a near-complete loss of particle mobility and the ubiquitous formation of bright, immobile puncta (Figure 2A). We then explored how the GC content of DNA affects condensation. AT-rich DNA initially condensed after the introduction of 5 μM spermine, followed by 20 μM spermine for DNA with a 57% GC content and 30 μM for GC-rich DNA (Table 1). The individual concentration of condensation in each DNA sample is determined by titrating in 5 μM increments until condensates form. No initial condensation is observed below the concentration shown in Table 1.

**Table 1:** Condensability of Different DNA Lengths and GC Content by Spermine

DNA length (bp)	spermine (4+)
70	50 μM
147 (57% GC)	20 μM
230	10 μM
AT rich (125bp)	5 μM
GC rich (125bp)	30 μM

The increased spermine-induced condensability of AT-rich DNA relative to GC-rich DNA confirms what was predicted from all-atom molecular dynamics simulations.^35^ The result is also consistent with the frequency of transient contacts between two double-stranded DNA molecules previously quantified by single-molecule FRET (smFRET).^35^ As we increased the length of DNA from 70 bp to 230 bp, the threshold concentration of spermine for DNA condensation decreased from 50 to 10 μM (Table 1), indicating that longer DNA molecules are easier to condense through intermolecular interactions mediated by spermine.

To further expand this study toward condensation in the chromatin context, we generated a mononucleosome construct containing a 147 bp 601 nucleosome positioning sequence (denoted as N) flanked by an 80 bp linker containing biotin and a 3 bp linker containing Cy5 wrapped around a histone octamer with a Cy3-labeled H2A histone (Figure 2B). This “80N3” nucleosome construct displays the same apparent two-dimensional diffusional behavior as naked DNA of the same length, and it can also form bright, immobile condensates upon addition of 100 mM spermine (Figure 2B). Using both 230 bp long DNA and 80N3 nucleosomes, we then tested several known and suspected condensing agents including polyamines, HP1α, and Ki-67 and determined the concentrations required to initiate condensation (Figure 2A, Table 2). Spermine (4+ valency) and spermidine (3+ valency) were both previously described to drive DNA condensation via electrostatic interactions.^50,39^ We found that these polyamines behaved similarly when condensing DNA and 80N3 (Figure 2B, Table 2); however, a lower concentration of spermine was required to initiate DNA condensation than nucleosomes, while the opposite is true for spermidine (Table 2). Interestingly, spermidine induced large, noncircular DNA condensates, where spermine at the same concentration, 100 mM (Figure 2C and 2D), only makes circular condensates. We also used the +2 charged polyamine putrescine and found that this was not sufficient to drive DNA condensation on the SLB even up to 1 M. We also tested HP1α, which binds to H3K9 methylated histones and promotes the formation of heterochromatin via recruitment of remodelers or binding partners.^3^ We found that a 5-fold higher concentration of HP1α was required to initially drive 80N3 nucleosome condensation than DNA condensation, which occurred at HP1α concentrations as low as 10 nM (Table 2). Definitive condensation by HP1α occurred at 150 nM for DNA and at 200 nM for 80N3 nucleosomes (Figure S2). Since HP1α has a DNA binding motif,^48^ our studies further suggest that improved DNA accessibility for binding by condensing agents leads to improved condensation. We also note that HP1α-mediated nucleosome condensation occurred even in the absence of H3K9 methylation. We did not observe condensate dissolution even at the highest concentrations of HP1α and Ki-67 tested (10 and 1 μM, respectively).

**Table 2:** Condensing Agent Concentrations Required To Initiate DNA or Nucleosome Condensation on a 2D Platform

	43N43 DNA	80N3 nucleosome
spermine (4+)	10 μM	20 μM
spermidine (3+)	30 μM	20 μM
putriscine (2+)	>1 M	>1 M
HP1α	10 nM	50 nM
Ki-67	1 nM	100 pM

### 3.2 Macromolecular Condensation Events Can Be Visualized at the Single-Molecule Level in Real Time

**Figure 3:**
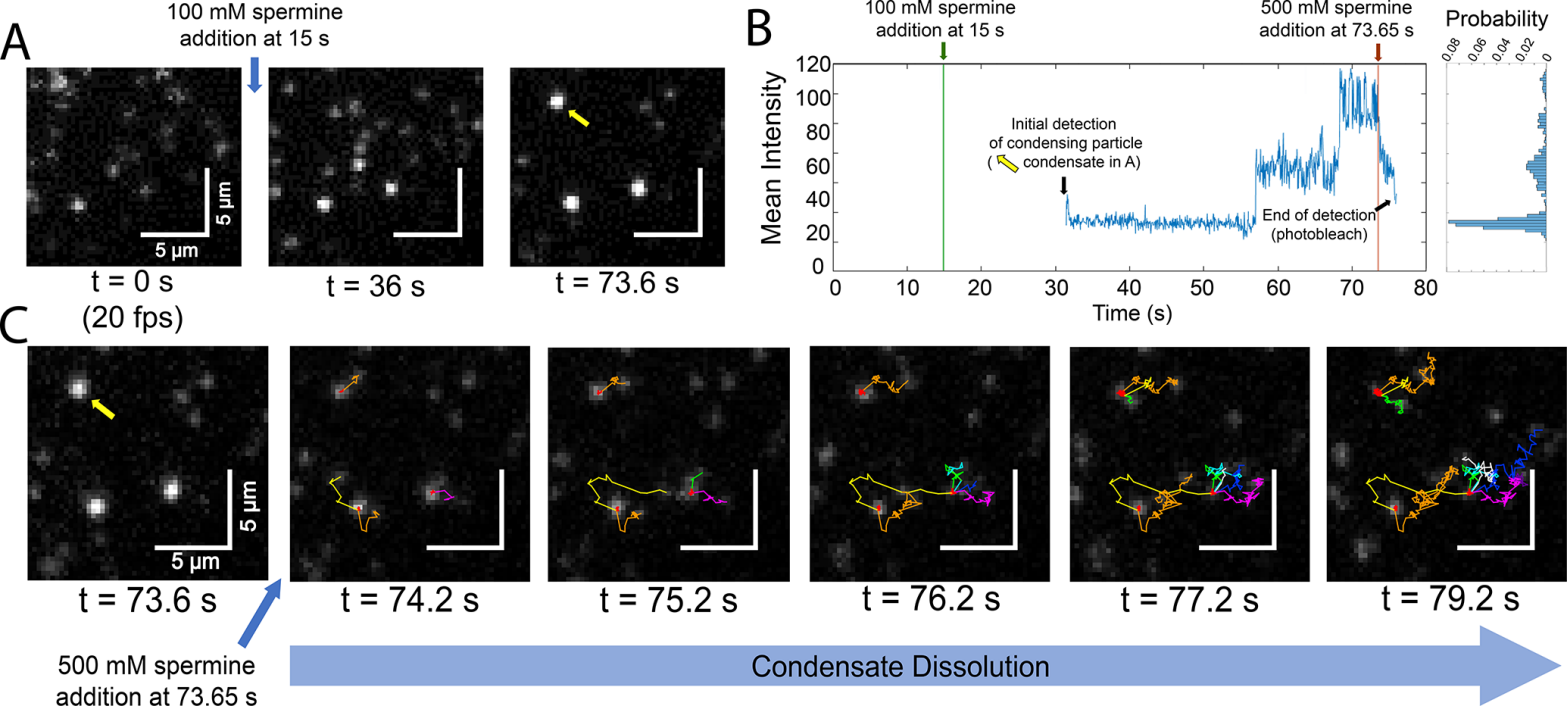
Real-time tracking of single molecules during condensation and dissolution. (A) Snapshots highlighting condensate formation after addition of 100 mM spermine. (B) Fluorescence intensity profile of a nucleating particle (denoted by a yellow arrow in A) during the inclusion of additional particles into the condensate. (C) Snapshots highlighting condensate dissolution after addition of 500 mM spermine to the SLB in A. New particle trajectories are given a different color.

Our SLB single-molecule imaging platform also allows us to visualize DNA and nucleosome condensation in real time. Single particles were tracked to determine their mean-squared displacement (MSD) and, subsequently, their diffusion coefficients. Cy3-labeled dsDNA bound to the SLB surface had an average diffusion coefficient of 0.49 ± 0.019 μm^2^/s with a lower limit of 0.001 μm^2^/s (Figure 1E). Single-particle diffusion coefficients were measured before and after addition of spermine, and a clear shift from a high-mobility state to a low- or no-mobility state was observed, from an average of 0.49 ± 0.019 to 0.17 ± 0.019 μm^2^/s with a shift back to 0.48 ± 0.025 μm^2^/s after the dispersal caused by spermine-mediated charge inversion (Figure 1E). A similar shift in diffusion coefficients was seen when subjecting 80N3 nucleosomes to the same spermine-mediated condensation, from an average of 0.94 ± 0.05 to 0.17 ± 0.019 μm^2^/s (Figure 2E). The condensate formation and dispersal pattern were not seen, however, when performing the same experiments on fluorescent particles that do not form condensates such as rhodamine-labeled phospholipids (Figure 2F). Real-time single-molecule experiments were carried out to visualize condensate formation and dissolution as the condensing agents were added via flow (Figure 3A). Once the condensates were fully formed, 500 mM spermine was added to promote condensate dispersal (Figure 3C), where we can see particles leave condensates and interact with other particles from dispersing condensates (Video 1, Figure 3C (orange trace)) or other condensates themselves (Video 1, Figure 3C (yellow trace)). From these videos, we were able to track the individual particles as they entered condensates by labeling each particle and observing their trajectories. We can then count the number of particles in a condensate by measuring the stepwise increase in intensity (Figure 3B), although transient interactions among condensate-forming particles coupled with photobleaching make it challenging to determine the number of particles per condensate reliably using intensity alone, and other metrics may be needed.

**Figure 4:**
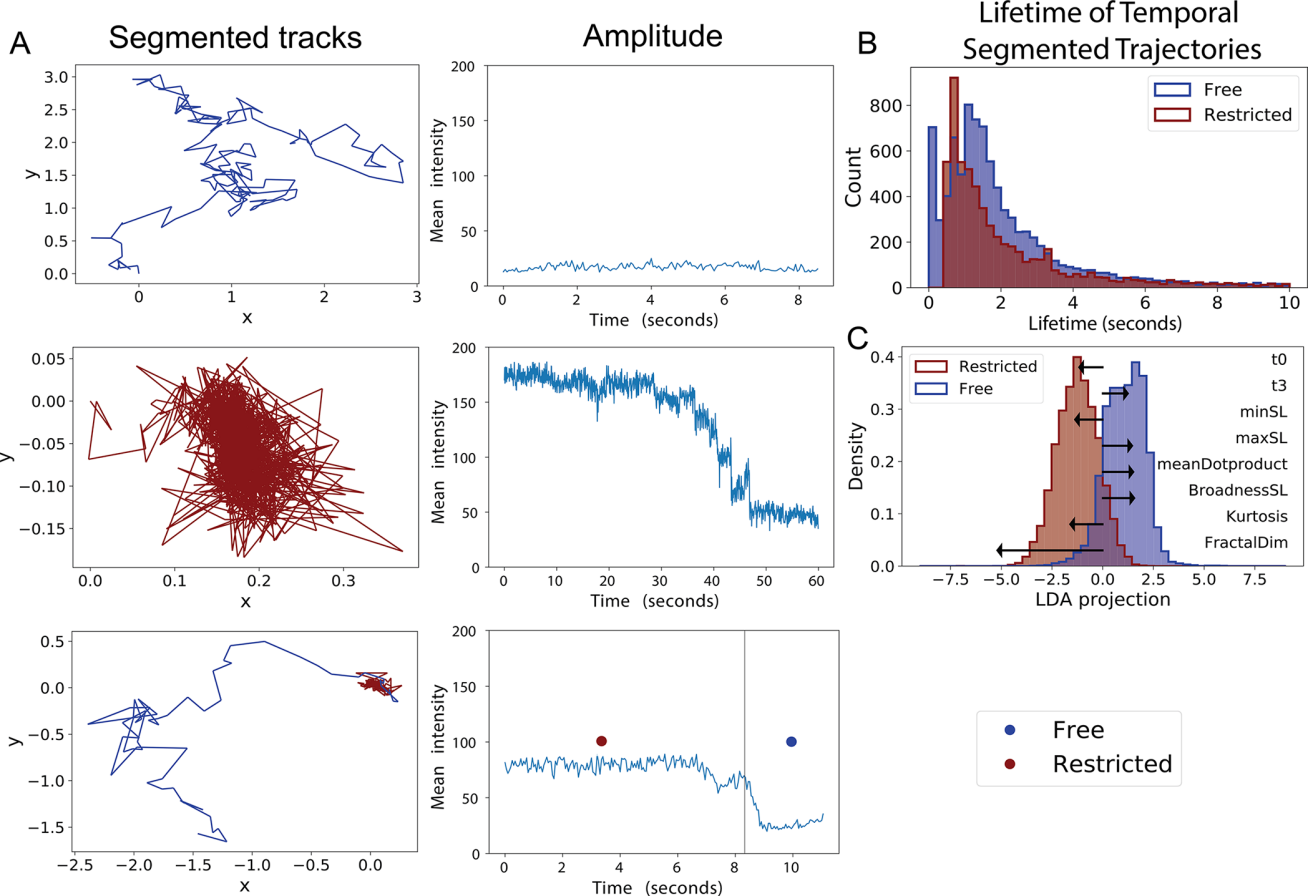
Diffusional fingerprinting of particles before and during dissolution. (A) Temporally segmented traces by rolling MSD analysis with their respective intensity profile, showing a representative free moving trace (top), restricted trace (middle), and heterogeneous diffusing trace. Intensity profiles show that the free moving tracks have a generally constant intensity amplitude, while the restricted trajectory has an intensity profile indicative of condensate disassembly. (B) Lifetime of free and restricted segments. (C) Linear discriminate analysis dimensionality reduced representation of the diffusional fingerprints of free and restricted trajectory segments with the top eight diffusional metrics separating the two distributions shown as black arrows. t0 and t3 describe residence times in the state with highest and lowest step length, respectively.

The large full-width half-maximum of the diffusion coefficient histograms seen in the diffusing particles (Figures 1E and 2E (blue histograms)) indicates that the particles tend to have heterogeneous diffusion behaviors, which can also be visualized through direct observation (Video 2), and highlights the multiple diffusion modes observed within a population of SLB-bound particles. To better track and categorize individual particles and their time-dependent diffusional behavior as they participate in condensation (Figure 4A), we utilized a rolling MSD analysis in conjunction with a machine-learning algorithm termed diffusional fingerprinting.^36^ From this analysis, we were able to categorize trajectories into subsegments of either a restricted or a free movement state and determine which diffusional state a particle spends the most time in (Figure 4B) and the specific diffusional metrics that differentiate these states (Figure 4C). In addition, we applied a fluorescence intensity analysis to observe a stepwise intensity increase during particle inclusion into condensates (Figure 3B) and during dissolution (Figure 4A). Interestingly, stepwise fluorescence intensity decreases can be seen when analyzing particles that have switched from restricted to free diffusion (Figure 4A, middle and bottom panels) which is also observed prior to dissolution. This intensity decrease is likely due to particles leaving a condensate or through photobleaching. With this diffusional fingerprinting analysis, we ranked the key distinct trajectory metrics that discriminate restricted movement, seemingly characteristic of condensates, from free moving particles. Using a linear discriminate analysis dimensionality reduction, we found that the fractal dimension of trajectories was the most important metric distinguishing “restricted” and “free” diffusional states (Figure 4C). The trajectory step length distribution, the span of distances particles travel in a frame, and kurtosis, a measure of distribution “tailed-ness”, are also both important in identifying condensing particles (Figure 4C). In addition, five other metrics made contributions (Figure 4C). When comparing the diffusion characteristics of trajectories collected over the course of 60 s, 28% of trajectories in the absence of spermine had more than one diffusion state vs 42% of trajectories in the presence of spermine (Table 3), likely due to particle inclusion into condensates.

**Table 3:** Temporal Diffusional Characteristics of 43N43 DNA before Inducing Condensation, after Condensation Is Induced, and When Condensation and Dissolution Are Initiated by Spermine Concurrently in the Same SLB as in Figure 3^a^

	particles with >1 diffusional state	particles with >2 diffusional states	particles with >1 diffusional state that end restricted	particles that are restricted their entire lifetime
precondensation	28%	14%	15%	16%
postcondensation	42%	26%	23%	29%

a Obtained from diffusional fingerprinting analysis and rolling MSD analysis where the diffusional states refer to “restricted” and “free”.

### 3.3 Ki-67-Driven Nucleosome Condensation

**Figure 5:**
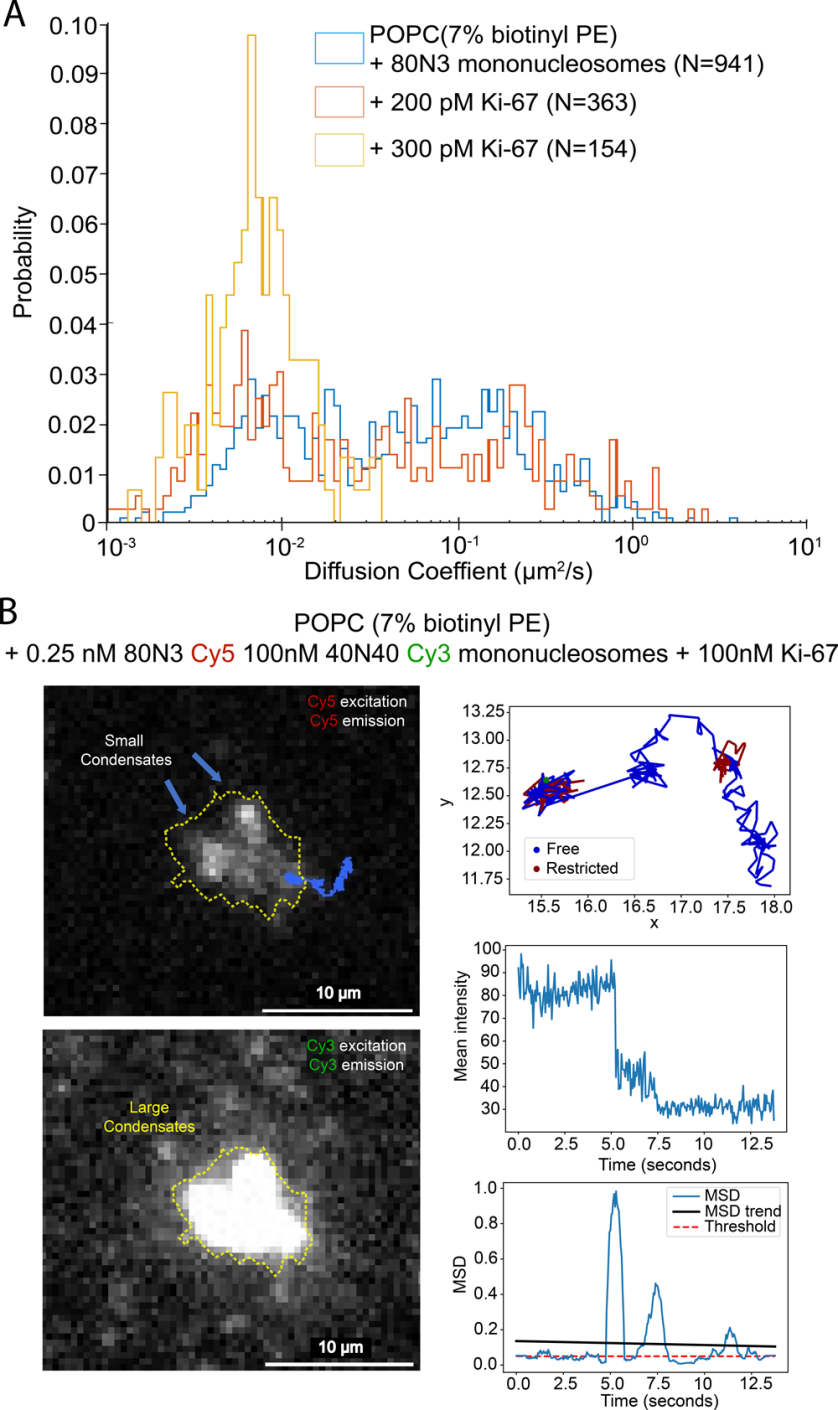
(A) Overlay of diffusion histograms obtained from tracked particles after addition of 200 and 300 pM Ki-67. (B) Large condensate sparsely labeled with Cy5 40N40 nucleosomes in a background of Cy3-labeled 80N3 nucleosomes. Condensate is outlined with a yellow dotted line, and trajectory of an interacting particle is highlighted in blue; intermediate-sized immobile condensates are shown using blue arrows (left panels). Trajectory specific diffusion/fluorescence intensity/rolling MSD profiles generated by temporal segmentation and diffusional fingerprinting (right panels).

We determined that Ki-67 is capable of condensing 1 nM mononucleosomes incubated on an SLB at as low as 300 pM Ki-67 concentration (Figure 5A). To investigate particle dynamics within Ki-67 condensates, we performed two-color experiments using 0.25 nM Cy5-labeled 80N3 nucleosomes and 100 nM Cy3-labeled 40N40 nucleosomes. This scheme allowed for the formation of large condensates that can be observed with Cy3 at low laser intensity, while the low concentration of Cy5-labeled nucleosomes allows for the tracking of single particles within and around the Cy3-verified condensates (Figure 5B). Borders of these Cy3-verified condensates are defined by a minimum fluorescence intensity of 90 (arbitrary unit) or triple the intensity of a single particle (Figure 5B (yellow borders)). Colocalized with these large condensates is a significant proportion of particles that are stationary as well as dense immobile condensates seen both by Cy3 and by sparsely labeled Cy5 (Figure 5B (blue arrows)), which implies that these large loosely packed condensates are composed of a network of the smaller, bright, dense condensates (Figures 2D and 5B) reminiscent of the dense bright condensates seen in previous experiments (Figure 2C). Visually, large condensates appear to take on a multitude of shapes, are immobile, and seldom show the circular shapes that are qualitatively associated with liquid–liquid phase-separated condensates. Nevertheless, particles are capable of associating with, dissociating from, and traveling within or through a Ki-67 condensate (Figure S3). Single-particle trajectories that associate with large condensates are observed by Cy5 emission to undergo sudden diffusional changes, seemingly as a particle stops interacting with a condensate (Figure 5B, S3). This is further supported by stepwise fluorescence intensity changes seen concurrently with sudden changes in a particle’s MSD (Figure 5B). These data suggest that after inducing condensation for 5 min, while these large condensates have static position and shape, they have undergone a phase transition while retaining some fluid-like properties associated with LLPS. Ki-67 condensates were not directly observed fusing to form larger condensates.

## 4 Conclusions

In this work, we have used supported lipid bilayers as a platform for visualizing the two-dimensional diffusion of surface-bound macromolecules such as DNA and nucleosomes in the presence of different condensing agents. We then observed that the threshold concentration of condensing agents depends on the length and GC content of the DNA, where DNA of greater length and lower GC contents led to initial condensation at lower condensing agent concentrations. Furthermore, DNA condensation by spermine can be reversed upon addition of a DNA saturating concentration of spermine, highlighting that the condensation does not perturb the SLB and allows for the observation of condensation and dissolution as reversible processes. We then used SLBs for visualizing real-time condensation and dispersal of condensates which allows tracking of individual particles entering and exiting condensates. These particle tracking data imply the presence of multiple diffusion states as indicated by the wide range of diffusion coefficients of DNA even in the absence of condensing agents. Using diffusional fingerprinting and temporal segmentation of diffusion we confirmed the existence of multiple diffusional states in DNA trajectories, namely, a mobile and immobile state, and identified their most discriminative diffusional characteristics.

We expanded this study by investigating the condensing behavior of nucleosomes in the presence of several known and suspected condensing agents such as Ki-67. Many proteins that participate in phase-separated condensates achieve condensation through multivalent interactions, where particles with a valency of 2 cannot form space-spanning interacting networks without linking to higher valence molecules.^16^ With evidence that full-length Ki-67 is capable of nucleosome condensation, it stands to reason that multiple motifs are likely responsible for Ki-67 condensation. To this end, future directions include testing the nucleosome condensability by individual Ki-67 motifs as well as investigating the impact of dephosphorylated Ki-67^37^ on nucleosome condensation.

Overall, this study serves to highlight the utility of SLBs as a tool for studying the real-time kinetics of nucleosome condensation and condensate dispersal, which can be further expanded to investigate other systems.^42^^55^ Membrane-bound systems are of particular interest,^29,56,44^ such as signal transducing biomolecular condensates that form as a result of membrane receptors binding their ligands.^46−47^

## Supporting Information Available

Widefield images depicting the fluorescence recovery after photobleaching (FRAP) of supported lipid bilayers as they are either bound by a macromolecule or not, oTIRF images highlighting the initial condensation of DNA and nucleosomes by HP1, and oTIRF/particle tracking analyses of particles as they interact with condensates (PDF)

## Notes

Video 1: Single particle tracking of SLB-tethered DNA during the charge inversion and dispersal of spermine-mediated DNA condensates. Video 2: Single molecule footage of 233 bp Cy3-labeled DNA bound to a supported lipid bilayer.

## Abbreviations

LLPS: liquid–liquid phase separation
SLB: supported lipid bilayer
SUV: small unilamellar vesicle
TIRM: total internal reflection microscopy
TIRF: total internal reflection fluorescence
MSD: mean squared displacement
LAP: linear assignment problem
LoG: Laplacian of Gaussian
FRAP: fluorescence recovery after photobleaching
FRET: fluorescence resonance energy transfer.
